# Community Mobilization for Slum Upgrading through Sanitation in Roma Informal Settlements in the Paris Region

**DOI:** 10.3389/fpubh.2017.00213

**Published:** 2017-08-21

**Authors:** Ipsita Nita Chaudhuri

**Affiliations:** ^1^American University of Paris, Paris, France

**Keywords:** participatory action research, sanitation, romani, environmental health, slum uprgrading, evaluation, community mobilisation

## Abstract

**Background:**

Community-based processes addressing environment, housing, and health issues may decrease health inequities by addressing social, economic, and political health determinants more effectively. Yet little analysis of their effectiveness has been undertaken and their potential transfer to marginalized groups in rich country settings. In Europe, stark health inequalities are seen among the Roma, Europe’s most impoverished community who often reside in informal settlements suffering from illiteracy, inadequate housing, and lack of water and sanitation. This paper assesses a dry sanitation project in a Roma informal settlement in the Paris region to improve their living conditions.

**Methods:**

Between 2014 and 2017, multiple stakeholders were involved in a participatory process of design, construction, and maintenance of toilets. Interviews, mapping, model construction, and facilitated discussion were used to identify design features and follow-up indicators. Field notes, videos, questionnaires, and observation provided data for monitoring and evaluation. For questionnaires delivered to women in the community, a cross section time series was conducted to due to migration.

**Results:**

Despite issues related to maintenance, the overall quality of life of women improved after toilet construction. This included indicators for comfort, cleanliness, practicality, privacy, security, and menstrual hygiene management. Furthermore, fewer women restrained themselves from relieving themselves or from drinking less water to avoid urinating. Odors continued to be an issue. Self-reporting of illnesses, such as diarrhea and urinary tract infections, were not reliable due to the vague description of these illnesses and the potential recall bias. Appropriate sanitation in informal settlements is a necessity as shown by feedback from Roma women and the literature. However, a more sustainable toilet project would have required an adequate budget, good quality materials, accountability, and buy in from all inhabitants as well as good relationships between the chief and all stakeholders in the settlement. Furthermore, provision should have been made for vulnerable and less autonomous inhabitants to ensure proper follow-up and maintenance.

**Conclusion/recommendations:**

Safe water and sanitation is essential to decrease health inequities. Formative research, identifying natural leaders, sanitation committee formation, outcome indicator and baseline questionnaire development, appropriate messages and toilet design and placement, long-term follow-up, and political support are elements for effective interventions with the Roma. Potential attrition and reporting bias should be considered in monitoring and evaluation.

## Introduction

Health inequities result from social stratification and systematic unequal distribution of power, prestige, and material resources among social groups. This shapes social hierarchies, creating conditions for social gradients in health. For migrants and ethnic minorities across Europe such as the Roma,^1^ the processes of social exclusion are considered a major cause of health inequalities (1, 2).

Among the most visible examples of health inequalities are those related to inadequate sanitation. In a project aimed at resolving one of the most extreme health inequalities facing a migrant community, local NGO’s built a set of dry toilets for an informal Roma settlement in the Paris suburbs. This paper describes this project and analyzes its development and outcomes over an 18-month period.

The Roma are one of the most impoverished and marginalized communities in Europe, often residing in informal settlements and labeled as “EU migrants.” Lack of access to water and sanitation is a central theme of their experience regardless of their immigration status, leading to negative health impacts (3).

Since the opening of Eastern Europe to the West many Roma have migrated to the region of Paris seeking access to health services, jobs, food security, and a better life. Several informal settlements have appeared with makeshift shelters made of flimsy materials that are flammable, contaminated, or rotting with mold. There is poor or no access to running water or sanitation.

## Background/Rationale

According to the WHO, water, sanitation and hygiene interventions (WASH) can reduce diarrheal diseases by 25–35%, and significantly reduce other water-related diseases (4, 5). In the WHO European region, 14 diarrhea deaths a day are estimated attributable to inadequate WASH. Other direct and indirect health outcomes have also been documented (6–10).

Centuries of Roma enslavement, the holocaust genocide, and subsequent systematic de-culturation by both western and communist governments after World War 2 have led to a distrust of outsiders (i.e., gadje) resulting in the Roma missing out on public health, sanitation, and literacy programs (11).

This marginalization has resulted in great health inequalities for the Roma through a nexus of extreme poverty, poor living conditions, lack of water and sanitation, little education, illiteracy, discrimination, poor access to health care, and lack of documentation (12–14).

Despite public health gains and the epidemiological transition for settled Europeans, the Roma, like other poor and minority groups, suffer a higher rate of chronic and acute/infectious disease than in more affluent groups (11). Yet, little quantifiable epidemiological data exist on the Roma because many Central and Eastern European countries do not collect ethnically segregated data. Health research on the Roma is consequently limited (15).

However, we know that life expectancy is at least a decade lower than for non-Roma (13). In addition, the Roma experience a higher infant mortality rate: 27.1 per 1,000 live births for non-Roma majority compared with 72.8 per 1,000 births for those of Roma ethnicity (15). The Roma population’s demographic pyramid resembles that of low-income countries with high reproductive rates and premature mortality leading to a high-density, child and low elderly population (13). Much like the developing world and emerging economies, the Roma suffer the double burden of diseases, as mentioned above, experienced by those who are poor and those that are affluent, such as obesity, diabetes, and cardiovascular disease (11, 15). Below, we discuss additional factors on the health of the Roma population.

### Roma and Informal Settlements

As a migratory group, without access to other forms of housing, the Roma often build makeshift accommodation without permission, on public or private land they do not own. They are, therefore, under constant eviction threat (14). The land is often marginal and contaminated, with sites near industrial facilities, waste dumps or old mining waste deposits increasing potential exposure to pathogens and chemical contamination (11, 14). Furthermore, because of the illegal and informal nature of their settlements, authorities have been reluctant to formalize services (14, 15).

### Tradition and Lifestyle

The Roma are often itinerant nomads, with a tribal organization and leader for each settlement (11, 16). Roma culture is very close-knit and based on patriarchal families. This creates large social capital and inter-family support. There is a fairly rigid division of male/female labor where women are expected to clean, cook, and keep caravans tidy (16). Traditions and customs are passed down orally in extended families (11, 16). Large extended families live in close quarters throughout Europe. Unfortunately, it is this overcrowding that can often provide an environment for the transmission of infectious diseases (11, 14, 15, 16).

To understand and work with the Roma, requires developing a respect for their organizational structure and their relationships to neighboring bands, accepting and respecting their gender division of labor, and working from the position of gadje to gain their acceptance (11, 16). Here, we first present factors to consider about their beliefs, education and schooling, language, and health status.

### Beliefs

The Roma are sensitive to spiritual and physical contamination from contact with dominant cultural groups. Their world is built around keeping their families uncontaminated from “gadje” (16).

The Roma have distinct beliefs regarding the purity of the top versus the bottom half of the body; the bottom half of the body is considered unclean and must be separated from direct and indirect contact with the top half, which is considered pure. Therefore, they are interested in internal/external cleanliness (11, 15, 16).

Traditional Roma concepts of purity or “Marime” are used to define complex social and physical boundaries. Contact with non-Roma and contact of upper body with lower body, intertwined with Marime and the dual concepts of good fortune or “Baxt” and bad luck or “Prikaza,” result in worldviews that associate contamination with bad luck (11).

Although the Roma have adopted religions based on where they have settled, a spiritual base exists for certain kinds of illness and for a belief in traditional curative remedies such as the power of spittle to treat wounds. This value system, combined with distrust of outsiders, deters the Roma from undergoing medical exams (11).

### Education and Schooling

Because of their mistrust of outsiders, many Roma do not consider schooling to be a priority. Although many have completed primary education they often do not go on to secondary or higher levels. Many girls and women in particular are illiterate (16).

#### Language

The Romani language is a key element of Roma identity. The language is a symbol of shared values, destiny, and nationhood and a link to their Indian origins (16).

### Health Status

Few cohort, case-controlled and case history studies exists for the Roma (11). UK travelers are the best documented. Studies of similar poor, minority status groups with migratory patterns of living, and working elucidate some of the health concerns relevant to Roma populations (11).

While hospital births are normal for the Roma, the use of preventative health services is low and most health-related issues are dealt with in emergency departments. Due to their family cohesiveness, many members of the family are often present during hospital visits (15). High rates of infant mortality are found among the Roma (11, 13).

Higher rates of sero-prevalence positive factors for the liver diseases hepatitis A, B, C, and E are found among the Roma compared to indigenous ethnic populations. Many studies have correlated high sero-positive antibody findings with crowded living conditions and a lack of piped water, sanitation, and waste handling (11).

The Roma migrating through Eastern Europe are also subject to communicable, animal and insect vector diseases, such as diphtheria, tick-borne encephalitis, lyme disease, yellow and typhoid fever, rabies, hepatitis A and B, cholera, and AIDS (11).

Immunization rates tend to be lower compared to the European majority in many European Roma groups. Major reasons for such differences may include limited information and medication identification, cultural beliefs concerning immunization, and/or reduced access due to mobility and economic status (11, 13).

Hungarian Roma have a high carrier frequency for a gene that has been found in high frequency in groups with resistance to the HIV virus (17). A case–control study in Spain of Roma school children showed prevalence of 92% HAV antibodies compared to 9% in non-Roma children suggesting immunity profiles differ from settled Europeans.

Alcohol abuse and illicit drug use is common, as is an increased risk of non-communicable diseases, such as diabetes and heart disease due to a genetic propensity to metabolic syndrome (15).

Some contend that Roma health status may be unrelated to being Roma but rather may result from their socioeconomic, cultural, or environmental conditions. For example, not attending regular health check-ups may be the result of barriers related to social exclusion, i.e., living far from service providers; discrimination that prevents Roma from accessing services without proof of residence; lack of health literacy due to multiple causes including exclusion from health education programs, illiteracy and a limited appreciation of the importance of prevention; cultural definitions of “ill” health, which discourage Roma engagement with health services; or lack of access to water and sanitation infrastructure (13).

Nevertheless, irrespective of whether the issues are specific to the Roma or more generic, improving Roma health outcomes requires a focus on disease prevention, health education, and promotional programs in areas, such as the environment, nutrition, physical activity, and alcohol/tobacco abuse. It also requires action in policy sectors, such as employment, education (including health and environmental education), housing, and access to safe water and sanitation. A thorough understanding of social, cultural, political, and behavioral factors is essential (3, 11, 13, 15).

The conditions found in European Roma settlements reflect those found in informal settlements globally. This paper describes a community mobilization process aimed at improving living conditions in a Roma settlement in a Parisian suburb by introducing dry sanitation. It examines the outcomes with a view to providing lessons learned.

#### The Case Study: Roma Informal Settlement in Triel sur Seine

The informal settlement in Triel sur Seine was created in 2009 with a rotating population of about 200 people, half of them children, comprising between 46 and 51 households. The settlement is in a field belonging to the city of Paris and private owners, situated out of public sight about 1 km from a major road. It was once a dump for sewage and industrial waste. Roma caravans on the site are often fifth hand from the Roma internal market and in ramshackle condition. Caravans are attached together, using bits of wood and plastic debris, to make common living quarters, making housing conditions deplorable. Water is accessible from a fire hydrant 1 km away. Inorganic and organic wastes are scattered throughout the camp mixed with reclaimed white goods, such as washing machines, televisions, and computers, which are sold by the Roma as scrap metal to recycling companies. Before toilet construction, inhabitants relieved themselves in the surrounding fields.

Several frameworks have been proposed to understand the construction, maintenance, and acceptance of dry toilet projects. However, they underrepresent the potential role of technology in influencing behavioral outcomes, focused on individual-level behavioral determinants largely ignore the role of the physical and natural environment. Dreibelbis’ integrated behaviour model (IBM) attempts to correct this by acknowledging three dimensions to analyze WASH: contextual (e.g., access to water and soap), psychosocial (e.g., shared values, perceived disease risks), and technological (e.g., availability and convenience of hardware). This will be used to frame the toilet project in this paper.

## Methods

Between 2014 and 2017, multiple stakeholders were involved in a participatory process of design, construction, and maintenance of dry toilets to be built on the site. Participatory methods, including interviews, mapping, model construction, and facilitated discussion, were used to identify design features and indicators to be followed before and after construction. Field notes, videos, questionnaires, and observation provided data for project monitoring and evaluation.

Several meetings were held with architects, public health professionals, NGO representatives from Caritas, the Roma chief, and men, women, and children of the informal settlement. At these meetings, successive drawings of toilet blocks were presented.

As a volunteer, the Principal Investigator (PI) was involved in guiding the process along with student architects for needs assessment, construction, monitoring, and evaluation by incorporating health, social, and environmental factors. This included elements, such as ventilation, cleaning, and maintenance protocols, to prevent the spread of pathogens as well as security issues.

As a paid health promoter, the PI also led discussions and workshops with women and adolescent girls focusing on issues of gender, sexuality, and reproduction. This work helped to identify appropriate elements in design, and construction. Specific focus group discussions (FGD) were also held on the subject of toilets. This resulted in the co-construction of questionnaires, incorporating inputs from site inhabitants, and external professionals, to track key impact indicators such as water consumption, cleanliness, safety, and disease incidence before and after toilet construction.

A mapping exercise helped to identify the appropriate toilet placement, taking into account water access and where the Roma defecated before toilet construction. To fulfill a social objective of the NGOs involved, French students were invited to participate in a construction weekend with the Roma and other volunteers.

Separate health education sessions were conducted with the community on infectious disease transmission, the importance of washing hands, and the proper maintenance of toilets for children, women, and the whole population. Given the high illiteracy rate in the community, illustrations, videos, and demonstrations were used to convey messages. Videos were made of the sessions in the Romani language, which were shown to other members of the settlement. Caritas volunteers and inhabitants also undertook regular monthly visual inspections and discussions to track progress of toilet use and material maintenance/degradation. This paper predominantly focuses on the results of surveys conducted with women to address their experiences; however, first some results are presented on experiences with toilet construction, relationships with the community throughout the process, results from focus groups discussions, and other relevant findings from the project.

## Results

### Community Relationships

The success of the toilet construction project was dependent on good contact and trust between the Roma chief, Caritas volunteers, and the architects. Dwellers in the settlement designated as their chief a man named Ion, as he was the first to have arrived in France 15 years earlier, with family members and neighbors from the same village in Romania migrating subsequently. However, a distinct disadvantage for subsequent follow-up of the project was that the chief did not himself live in the slum. There was no accountability to him for the proper maintenance of some toilet cabins.

Furthermore, the relationship of the chief with the rest of the community was tenuous and hinged upon his involvement and support of the evangelical church that was built on the informal settlement. Factions had developed in the settlement as a result with other familial and social conflicts. This had made accountability and managing the toilets difficult.

#### FGD and Outcomes in Construction Design

Although slum dwellers belonged to extended families, during focus groups discussions women and men insisted that toilets could not be shared between different family factions. Individuals did not want to clean the toilets of others. Roma taboos exist around the purity of the top of the body considered pure and clean versus the bottom half, which is considered unclean. According to the Roma they must be separated from direct and indirect contact and, therefore, related to internal/external cleanliness (11, 15, 16). This required the construction of separate cabins for each family (normally mother, father, and children which at times included grandparents). Using a mapping exercise, slum dwellers identified a distance of 30 m collectively as appropriate between caravans and toilet blocks.

**Figure 1:**
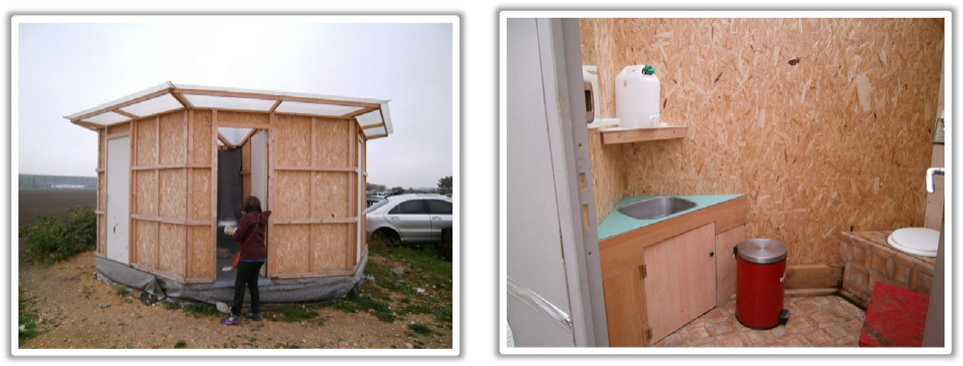
Toilet blocks.

In order to accommodate each family in the slum, five blocks of six toilet cabins were constructed, with five Roma men identified by the chief to be in charge of each structure. Each block had a common pit dug that was shared by all the cabins in the block (Figure 1).

As a volunteer and public health specialist in toilet design and construction, I facilitated discussions to resolve conflicts between esthetic design objectives and budget constraints for the architects, practical considerations for the Roma arising from the sharing of toilet blocks and pits, and public health considerations (pathogen exposure).

Architects, students, and the Roma worked side by side to construct toilets for one weekend. After which anything not finished was left for the Roma to complete themselves. This included ventilation pipes, pit encapsulation, and the double wall for weatherproofing. A Roma inhabitant who was a mason and an architectural student finished the encapsulation and installing ventilation pipes and mechanical fans. However, the weatherproofing wall was not finished on the external structure as well as interior finishing. These were left to the slum dwellers.

Responsibility for these toilets was given solely to the Roma after construction. However, the illegal tenure of the settlement and lack of a formal decision-making structure complicated accountability and poor maintenance resulted in problems of potential pathogen spread. Social cohesion was variable and factions did occur between groups in the camp.

Because of a variable commitment to finishing the toilets as well as limited construction abilities of the inhabitants and very low disposable incomes, toilet completion was inconsistent. Subsequently, toilet cleanliness and maintenance was also uneven. For those households in which male heads did not support toilet use, little effort was made to ensure finished toilets. In addition, their wives who were responsible for toilet cleanliness invested little time and effort. Many of these men were unable to obtain employment and were often seen under the influence of alcohol. Children often defecated on cabin floors which were not cleaned regularly. This could have potentially contributed to pathogen dispersal.

Several families have been rehoused since the project and new settlers have taken these toilets. Others have been left in disuse.

#### Other Results and Findings

##### Design and Quality of Building Materials

Due to a limited budget, building material quality was poor. This resulted in the materials weathering, deteriorating, and collecting mold. Roofs often were displaced due to violent storms during the winter. Toilet seats were often broken and not replaced or fastened. When women urinated, urine escaped through the bottom of the seat structure and sprayed on the ground in front of them. This made cleaning difficult and a lining had to be put in place by the Roma.

Odor and ventilation continued to be an issue. This was in part because sawdust was not used to absorb liquid and odors. The Roma did not want to make the effort to source this sawdust, which required petrol to drive to the sawmill. The common pit between the occupants of six cabins meant that there was little control of what was deposited affecting the other cabins. Women, therefore, used fresheners to mask the smell.

##### Vandalism

Vandalism was problematic as water containers; soap holders, locks, and toilets seats were often broken. However, little vandalism occurred in cabins where the male responsible for the toilet structures was proactive.

##### Migration

Due to the relocation of many Roma families during the course of the year, toilets changed hands and, therefore, it was difficult to follow the impacts of the toilet on each family. Many women who did not have toilets did not have male household heads and were single parent families. They were never allocated toilets.

#### Survey Results with Women

Despite issues related to maintenance and follow-up, an overall improvement in the quality of women’s lives after toilet construction was identified by indicators collected three times (pre- construction, 3 months post- and 18 months post-), which are discussed below and shown in Table 1. Given the migratory nature of the population, a time-series cross-sectional population survey was carried out with a *n* = 30. The average number of inhabitants in the camp including men, women, and children was 200.

**Table 1:** Monitoring and evaluation indicators of toilet construction particularly relevant to women and children.

	BC yes July 2015 (*n* = 30) (%)	AC yes October 2015 (*n* = 30) (%)	AC yes December 2016 (*n* = 30) (%)	BC No July 2015 (*n* = 30) (%)	AC No October 2015 (*n* = 30) (%)	AC No December 2016 (*n* = 30) (%)
Does the absence of toilets stop you from relieving yourself when you need to?	100	88	56	0	12	44
Do you drink less to avoid going to the toilet?	85	36	39	15	64	61

When you go to the toilet is it						

Practical	20	91	72	80	9	28
Comfortable	12	92	61	88	8	39
Clean	22	96	72	78	4	28
Do you find the odor of urine and excrement a nuisance when you go to the toilet?	89	83	67	11	17	33
Is the absence of water a problem when you relieve yourself?	96	88	56	4	12	44
Do you wash your hands after going to the toilet?	100	95	100	0	5	0
Do you have access to water/soap/disinfectant when you wash your hands?	50	75	56	50	25	44

Did your children have the following illnesses the week before						

Diarrhea	30	9	6	70	91	94
Urinary tract infection	15	0	6	85	100	94
Intestinal parasite	4	4	12	96	96	88
Eye infection	8	9	6	92	91	94
Is it difficult to manage your menstrual hygiene problems when you go to the toilet?	92	29	53	8	71	47
Is Privacy a problem for you when you go to the toilet?	96	25	41	4	75	59
Do you feel safe when you go to the toilet?	15	92	71	85	8	29

*BC, before construction, AC, after construction*.

##### Monitoring and Evaluation Indicators Relevant to Women and Children

Focus groups discussions with women and adolescent girls highlighted several disadvantages of defecating in the surrounding fields. They included, lack of privacy, security, and difficulty in menstrual hygiene management. In addition, women prevented themselves from drinking too much to avoid going to the bathroom or delayed going due to inclement weather and darkness. Relieving oneself in the field was also considered not clean, practical, or comfortable. These elements were used to guide toilet placement and structural design. In addition, a questionnaire was developed with the Roma and administered with Roma adolescents to track these indicators 2 weeks before and 3 and 18 months after construction.

##### Hygiene

After the construction of the toilet, the percentage of women who said that they clean their hands after using the toilet increased and fell slightly 18 months later. However, access to water continued to be a problem as the only access point was 1 km away at a hydrant in a cemetery. Many women walked to this access point with make shift wheelbarrows or strollers to fill containers for daily use. Soap and disinfectant use increased 3 months after construction but decreased 18 months later.

Among those women who did not use toilets, there was a perception that defecating in the fields was cleaner.

##### Menstrual Hygiene Management

Before the construction of toilets, 92% of women said that they had trouble managing their menstrual hygiene needs. After the construction there was a substantial decrease to 29%, followed however, by an increase to 53% 18 months later. This increase in the third survey was at least in part related to the inclusion of women who did not have a toilet block allocated to them as they had come to the camp after the toilets had been assigned.

##### Illnesses

Self-reporting of childhood illness 1 week before questionnaire administration revealed that 30% of households had one child or more with diarrhea before construction. This decreased to 9 and 6% in the second and third questionnaires. While very few respondents noted urinary tract infections (15%), intestinal parasites (4%), or eye infection (8%), however, a decrease was reported in the second and third surveys.

These figures, however, are not reliable because of the vague description of these illnesses and the potential biases in self-declaration. During the year after construction of the toilet a Hepatitis A (a fecal oral disease) outbreak occurred and a subsequent immunization campaign was implemented with the distribution of chlorine to encourage toilet maintenance and cleanliness. However, it was not possible to establish the relationship between toilet construction, maintenance and illnesses because the numbers surveyed were too small to conduct a statistical analysis.

##### Privacy and Safety

Ninety-six percent of women felt that privacy was a problem when relieving themselves in the field while 85% felt that safety was an issue before construction. After construction of the toilets, only 25% felt privacy and 8% felt safety to be issues. Eighteen months after the construction, baseline privacy and safety concerns rose with 41% saying privacy as an issue and 71% saying safety was an issue. In interpreting these figures, it should be noted that seven (23.3%) of the women in the third survey were not allocated toilets and still relieved themselves in the fields.

The lack of toilets before construction discouraged women from relieving themselves. This reluctance diminished slightly 3 months after construction and at was reported by at least 50% fewer 18 months later. Before the construction, 85% of women surveyed said they drank less in order to avoid relieving themselves. Three months after construction, 36% of women said they drank less. This percentage remained relatively similar at 39% 18 months later. Before construction of toilets, 20 and 22% of women, respectively, said that relieving themselves was practical and clean, while only 12% said that it was comfortable. These values rose substantially to above 90% for all three indicators 3 months after the construction. Eighteen months these numbers dropped to 72% for practical and clean and 61% comfortable. Odor was considered a nuisance before the construction of toilets (89%) and continued to be so after (83%).

The absence of water when women relieved themselves was considered a great problem before construction at 96% and stilled continued to be a problem 3 months later at 88%. However, only 56% of women considered it to be a problem 18 months later.

More than 90% of women said they washed their hands when relieving themselves before construction of the toilets. This number rose to 100% 3 months after construction and remained only slightly less 18 months later. The availability of soap and disinfectant was a problem even after construction.

## Discussion and Conclusion

Appropriate sanitation in informal settlements is a necessity as shown by feedback from Roma women and the literature. However, good toilet construction and maintenance required an adequate budget, accountability, and buy in from decision makers in the settlement. Furthermore, provision should have been made for vulnerable and less autonomous inhabitants to ensure proper follow-up and maintenance.

The design, construction, and maintenance of the toilets were dependent on good relations and trust between the camp chief, and “gadges” (outsiders), including caritas volunteers, architects, and public health specialist. Although this toilet project engaged the Roma in the design and construction of the toilets, the ownership of the process by inhabitants was variable. This was due to variable social cohesion and factions which existed between groups in the camp. This made accountability and maintenance difficult.

Responsibility for these toilets was given solely to the Roma after construction; however, given the Roma have illegal tenure and no formal decision-making structure this also made accountability difficult. Little disposable income to obtain materials to finish the construction and for maintenance increased the potential spread of pathogens.

Health indicators were difficult to track, as clinical and microbiological data were not collected. When a hepatitis A outbreak occurred in the settlement which is typical of the crowded conditions found in Roma settlements, it was difficult to attribute this to toilet maintenance.

An analysis of this WASH experience is provided below using Dreibelbis integrated behavior model, which includes not only behavioral determinants but also the role of the natural and physical environment and technology (Table 2). This is followed by recommendations for sanitation interventions for NGO’s, local authorities and multi-lateral institutions based on the lessons learned in this project.

**Table 2:** Integrated behavior model for WASH for the Roma in Paris Region.

Levels	Contextual factors	Psychosocial factors	Technology factors
Societal/structural	Water and sanitation are a right internationally; however, local legal frameworks supersede (3)	Leadership-Agreement of male leader essential. Roma camps in the Ile de France each have a male leader who is often the one that has first arrived in the area	Manufacturing, financing, promotion, and distribution of products
	The Roma have settled on land for which they have no legal tenure. Different parcels of the land have municipal, corporate, and private owners	Cultural identity—Roma identify within their social networks and cohesion in the community creates social pressure to adopt appropriate hygiene practices	Materials were of low quality and did not wear well during the winter months. Continual rain led to leaks and potential mold build up and required continual maintenance
	Average climate, sunshine, rainfall, geography—absorption and porosity needs to be determined	Trust of the outsider is very important as the toilet projects was brought in from outsiders or “gadge”	Financing is often available through NGOs. No continued support
	Average Climate the Ile de France—Oceanic and continental weather		The cost of pre-fabricated toilets is prohibitive given Roma poverty and a community-led sanitation approach where design was dependent on the Roma needs. This type of toilet, however, would have been more durable
	Average temperature: 19.5°C summer and 5.0°C winter		
	Average sunshine: 1798 h per year (Paris)		
	Average rainfall: Summer—52.4 mm Winter—50.5 mm equivalent to 641.6 mm per year (Paris)		

Community	Access to markets, access to resources, built and physical environment.	Shared values, collective efficacy, social integration, stigma Roma live in community so social norms and the acceptance of practices should be related to these norms	Location, access, availability, collective ownership, maintenance

Interpersonal/household	Roles, household structure, division of labor, available space Strict division of labor between male/female Separate consultation for men and women Men in charge of construction Women responsible for cleaning Crowded caravans children spend most of their time outside and as do adults when socializing. Small children urinate and defecate freely? Mothers not always attentive to the hygiene needs and safe disposal of feces?	Norms, aspirations, shame, nurture Purity and cleanliness are important aspects of Roma beliefs Shame related to the lower part of the body which cannot touch the upper half of the body.	Access to product, demonstration of use of products

Individual	Wealth, age, education, gender, livelihoods	Self-efficacy, knowledge, disgust, perceived threat	Perceived cost, convenience, strength, and weaknesses of product
		Although disgust of fecal matter is a part of Roma culture. This was not used to convince the Roma to build toilets	The Roma were not willing to drive to obtain sawdust to absorb liquid and odors.
		Organic and inorganic solid waste debris is found all over the camp and particularly around toilet structures. Little effort is made to clean this on a regular basis and it did not appear that the Roma perceived this as an issue	Odors remain a problem and some say there are less odors in the fields

Habitual	Facilitators/barriers to habit formation Low literacy levels (particularly of mothers) Is there buy-in from leader of settlement? Cohesion in the settlement is varied and a number of factions exist within it making accountability and maintenance of the toilets uneven?	Existing water and sanitation habits, outcome expectations Information on anal cleansing habits are important in order to determine what types of hand washing messages need to be passed. In addition, the provision of water in toilets is necessary	Ease and effectiveness of routine use of product Roma don’t want to share toilets with other families?

## Recommendations

The following are recommendations for toilet construction in Roma settlements for local authorities, public health departments, NGO’s and multilateral institutions.

- Ensure access to safe water and sanitation for the Roma
- Formative research
  - Identify sanitation needs and construction feasibility through formative research.
  - Use IBM framework to frame research with a well-trained facilitator.
  - Consider location, informal settlement variability, and population mobility for onsite sanitation.
  - Determine if toilets can be properly maintained to avoid contamination.
  - Use FGD with convenience samples to take into consideration in and out migration.
  - Include stakeholders such as municipal authorities, NGO’s, and health agencies.
- Natural Leader identification
  - Identify Roma leader for settlement.
  - FGD should be held separately between women, men, young adolescent girls, and children.
- Potential Themes for FGD
  - Safety—is personal safety an issue? Where is a safe location for defecation?
  - Privacy—how can privacy be ensured?
  - Security—are locks necessary?
  - Comfort—what is the preferred defecation position?
  - Anal cleansing practices—intermediary substance (paper, leaves etc.) or hands with water or both will determine the importance of cleaning with soap and water after defecation. This is private and morally bound issue and needs to be asked with care and discretion (18).
  - Child stool disposal–
  - Local practices, perceptions of illness causes, tradition, and taboos.
- Formation of Sanitation Committee
  - Determine feasibility of sanitation committee dependent on the nature of movement in settlements and buy-in from leaders.
  - Identify if cohesion exists in informal settlements
  - Ensure long-term follow-up by committee and social pressure on the community for ownership, pride, and collective efficacy in maintaining a clean settlement.
  - Identify community norms to support proper construction, maintenance, and behavior.
- Development of Outcome Indicators
  Identify social and health indicators
  - Hygiene behaviors (handwashing with soap, child feces disposal in potty or latrine, cleaning child’s bottom, soap presence in latrine)
  - Social indicators (prestige, self-respect, privacy, safety, security, comfort)
  - Technical indicators (cleanliness, functionality).
  - Health outcome indicators (diarrhea incidence, intestinal worm infections) and anthropometric measures (weight for height, height for age, and the prevalence of stunting). Some studies have even examined impacts on trachoma (19).
  - Take into consideration epidemics, climate fluctuations, vaccination status, nutritional status, environmental enteropathy when developing indicators (18, 20)
  - Obtain an epidemiological profile of prevalent sanitation-related diseases in home countries, such as hepatitis or intestinal worms. This may identify sanitation related diseases not normally present in host country.
  - Identify hygiene behaviors through transect walks and FGD
  - Measurements of biological pathogens in latrine areas may provide relevant information on cleaning and maintenance practices and potential exposure pathways
- Development of baseline questionnaire
  - Use cross-sectional study design for four different seasons in a Roma informal settlement. The differences in the characteristics of the individuals due to attrition and losses to follow-up due to in and out migration should be noted.
  - Translate questionnaires, interviews into native languages including Romani to mitigate reporting and recall bias.
  - Work with children as drivers of change and potential translators and interpreters.
- Development of Appropriate Messages
  Targeted messages, using enough channels and attractive appropriate materials are essential for the successful health and environment education (20).
  - Determine how the notions of purity and cleanliness between the upper and lower part of the body for the Roma fit into this messaging using emotive factors, such as shame and disgust in CLTS (11, 15, 20, 21).
  - Work with Roma children as change agents to develop educational materials that are appropriate and attractive along with their young mothers who do not speak host country languages and are mostly illiterate. This may be a way to develop appropriate and attractive engaging visuals.
- Appropriate toilet design and placement
  - Architects and planners can use the participatory hygiene and sanitation transformation, water and sanitation mapping exercise to identify appropriate toilet design and placement.
  - Identify appropriate building materials for cost, cleaning, and maintenance
  - Identify soil conditions, absorption capacity, and space for temporary structures (22).
- Long-term follow-up
  - Use Roma oral networks in Romani to sustain messages and provide technical advice and soft skills necessary for safe disposal of feces.
  - Ensure sanitation-related infectious diseases are not spread in urban environment to other citizens (11).
  - Identify correct health indicators. For example, is diarrhea an important indicator for children under 3 years old? Is it relevant in developed settings such as those found in European cities where the water supply is potable and access to medication may be more available than in low-income countries?
  - Determine effective monitoring of toilet use, appropriate design, hygiene behavior, and changes in health equity that takes into consideration migration.
  - Ensure on-going communication with leaders Europe.
  - Understand the importance of environmental enteropathy in children due to contaminated environments and how this might influence gut absorption and growth using biomarkers.
  - Explore potential immunity to particular types of pathogens.
- Political Support- Macro-structural Factors

Support local, regional, national, and international level for the long-term prevention of sanitation related diseases in Roma informal settlements.

- Protect Roma population from infectious diseases
- Protect surrounding populations from epidemics of sanitation related diseases
- Provide subsidies for toilets and hygiene behavior programs that are well conceived and well executed.
- Provide qualified staff to monitor and provide long term follow-up of the sanitation situation of this migrant population and their hygiene behaviors.
- Provide water in order for effective sanitation programs to take place, therefore, adequate and accessible water provision is a priority for these Roma informal settlements.

An ethics review process was not required:

This research was carried out as outreach work in a volunteer capacity with Caritas Secours Catholique. Notes and questionnaires were taken to ensure the proper functioning of toilets and to provide recommendations. The notes formed the basis of the article.

Caritas Secours Catholique has an ethical code of conduct that is signed by every volunteer, therefore, no ethics approval was required for this activity. No ethics approval was required per institutional regulations and national guidelines.

## Author Contributions

IC is the principal researcher and investigator in this project.

## Conflict of Interest Statement

The author declares that the research was conducted in the absence of any commercial or financial relationships that could be construed as a potential conflict of interest.

## Footnotes

^1^The Roma population totals 10–12 million in Europe.
